# Organoid models to predict chemotherapy response and guide surgical strategy in pancreatic cancer

**DOI:** 10.1097/MS9.0000000000004345

**Published:** 2025-11-14

**Authors:** Hasibullah Aminpoor, Muhammad Zaib, Muhammad Khizar, Marhama Alamgeer, Hasiba Karimi

**Affiliations:** a Faculty of Medicine, Kabul University of Medical Sciences “Abu Ali Ibn Sina”, Kabul, Afghanistan; b Faculty of Medicine, Georgian American University, Manifest Medical Research Co, Tbilisi, Georgia; c Faculty of Medicine, Shaheed Mohtarma Benazir Bhutto Medical College Lyari, Karachi, Pakistan; d Faculty of Medicine, Bezmialem Vakif University, Istanbul, Turkey

**Keywords:** adenocarcinoma, chemotherapy, pancreatic cancer, surgery

## Abstract

Pancreatic ductal adenocarcinoma (PDAC) remains one of the most lethal malignancies, with limited predictive biomarkers for chemotherapy response. Patient-derived organoids (PDOs), developed from endoscopic or surgical samples, faithfully replicate tumor genetics and microarchitecture, allowing rapid *ex vivo* drug testing. Early studies from the United States, Japan, and Europe demonstrate that PDO chemosensitivity strongly correlates with clinical response, enabling personalized preoperative strategies. PDO-guided chemotherapy could refine neoadjuvant selection, minimize ineffective regimens, and inform optimal surgical timing. This translational advance represents a promising shift toward precision surgery in PDAC, warranting international standardization and integration into multidisciplinary workflows.

*To the Editor*,

Pancreatic ductal adenocarcinoma is the seventh leading cause of cancer death globally, with a 5-year survival of less than 12%^[1]^. Despite advances in multimodal therapy, the heterogeneous response to chemotherapy continues to undermine outcomes. Neoadjuvant therapy improves R0 resection rates, yet clinicians lack reliable tools to predict who will benefit. Organoid-based models now offer a means to preoperatively assess tumor-specific chemosensitivity.

Patient-derived organoids (PDOs) are three-dimensional tumor cultures derived from patient biopsies or surgical specimens that preserve the genetic and morphological features of the primary tumor^[2]^. Their establishment success exceeds 75% even from small endoscopic ultrasound-guided biopsies^[3]^. *In vitro* drug testing on PDOs can identify individualized response patterns within weeks, enabling pre-surgical chemotherapy optimization. Notably, Tiriac *et al* first demonstrated that PDO drug sensitivity correlates with clinical outcomes, providing proof-of-concept for personalized therapy^[4]^.

Recent international evidence reinforces this potential. In the United States, Demyan *et al* confirmed that PDO profiles predict neoadjuvant response and correlate with histopathological regression scores^[1]^. In Japan, Oyama *et al* established PDOs from Endoscopic Ultrasound–Guided Fine-Needle Aspiration (EUS-FNA) samples and identified transcriptional signatures of gemcitabine and paclitaxel resistance^[5]^. European trials report similar success integrating PDO-guided selection into precision oncology frameworks^[3]^. Collectively, these studies suggest that preoperative PDO screening could guide regimen choice, avoid ineffective therapy, and tailor surgical timing.

The translational implications are substantial: PDO-guided testing could transform the neoadjuvant phase from empiricism to evidence-based precision, improving survival while sparing patients unnecessary toxicity. As this technology expands, transparent and standardized reporting of associated computational analyses, particularly those involving artificial intelligence, is essential.

Our work is in line with the TITAN Guidelines on the need for transparency in AI use in healthcare^[6]^.

## Data Availability

Not applicable.

## References

[1] Demyan L Habowski AN Plenker D. Pancreatic cancer patient-derived organoids can predict response to neoadjuvant chemotherapy. Ann Surg 2022;276:450–62. 35972511 10.1097/SLA.0000000000005558 PMC10202108

[2] Xu J Pham MD Corbo V. Advancing pancreatic cancer research and therapeutics: the transformative role of organoid technology. Exp Mol Med 2025;57:50–58. 39814914 10.1038/s12276-024-01378-w PMC11799150

[3] Geny P Agostini A Larghi A. Pancreatic cancer patient-derived organoid platforms: a clinical tool to study treatment response. Front Med (Lausanne) 2021;8:793144. 35004765 10.3389/fmed.2021.793144 PMC8733292

[4] Tiriac H Belleau P Engle DD. Organoid profiling identifies common responders to chemotherapy in pancreatic cancer. Cancer Discov 2018;8:1112–29. 29853643 10.1158/2159-8290.CD-18-0349 PMC6125219

[5] Oyama S Matsuda A Murakami R. Pancreatic cancer organoids derived from EUS-guided FNA specimens can be used to predict chemotherapy resistance. Sci Rep 2025;15:23818. 40610535 10.1038/s41598-025-09395-z PMC12229655

[6] Agha RA Mathew G Rashid R. Transparency in the reporting of Artificial INtelligence – the TITAN guideline. Prem J Sci 2025;10:100082.

